# The transcriptional landscape of *Rhizoctonia solani* AG1-IA during infection of soybean as defined by RNA-seq

**DOI:** 10.1371/journal.pone.0184095

**Published:** 2017-09-06

**Authors:** Tanya R. Copley, Raj Duggavathi, Suha Jabaji

**Affiliations:** 1 Plant Science Department, McGill University, Ste-Anne-de-Bellevue, Quebec, Canada; 2 Animal Science Department, McGill University, Ste-Anne-de-Bellevue, Quebec, Canada; Leibniz-Institute of Vegetable and Ornamental Crops, GERMANY

## Abstract

*Rhizoctonia solani* Kühn infects most plant families and can cause significant agricultural yield losses worldwide; however, plant resistance to this disease is rare and short-lived, and therefore poorly understood, resulting in the use of chemical pesticides for its control. Understanding the functional responses of this pathogen during host infection can help elucidate the molecular mechanisms that are necessary for successful host invasion. Using the pathosystem model soybean-*R*. *solani* anastomosis group AG1-IA, we examined the global transcriptional responses of *R*. *solani* during early and late infection stages of soybean by applying an RNA-seq approach. Approximately, 148 million clean paired-end reads, representing 93% of *R*. *solani* AG1-IA genes, were obtained from the sequenced libraries. Analysis of *R*. *solani* AG1-IA transcripts during soybean invasion revealed that most genes were similarly expressed during early and late infection stages, and only 11% and 15% of the expressed genes were differentially expressed during early and late infection stages, respectively. Analyses of the differentially expressed genes (DEGs) revealed shifts in molecular pathways involved in antibiotics biosynthesis, amino acid and carbohydrate metabolism, as well as pathways involved in antioxidant production. Furthermore, several KEGG pathways were unique to each time point, particularly the up-regulation of genes related to toxin degradation (e.g., nicotinate and nicotinamid metabolism) at onset of necrosis, and those linked to synthesis of anti-microbial compounds and pyridoxine (vitamin B6) biosynthesis 24 h.p.o. of necrosis. These results suggest that particular genes or pathways are required for either invasion or disease development. Overall, this study provides the first insights into *R*. *solani* AG1-IA transcriptome responses to soybean invasion providing beneficial information for future targeted control methods of this successful pathogen.

## Introduction

Rhizoctonia foliar blight (RFB) or aerial blight first appeared in Louisiana, U.S.A. in the 1950s on soybean (*Glycine max* (L.) Merr.) [1]. Recent outbreaks of the disease in Brazil and in the southern states of the U.S.A. have caused yield losses of 30–60% [2–4]. This diseases is caused by rain splashed soils containing sclerotia and mycelial fragments (asexual stage) that grow along the plant surface, eventually reaching the upper portion of the plant and spreading from leaf to leaf and plant to plant. RFB occurs in high humidity and temperate environments, and is most destructive following canopy closure and during seed pod development [5]. RFB of soybean is difficult to control as labeled fungicides and the use of less-susceptible cultivars has limited effectiveness [6–8]. Isolates of *Rhizoctonia solani* causing RFB have been characterized as anastomosis group (AG) 1 intraspecific group or subgroup IA [9] with populations that are associated with Fabaceous hosts (soybean), and others that are taxonomically related, yet genetically distinct and infect Poaceae hosts (e.g. rice) [4, 10].

The sheath blight disease (SBD) of rice caused by *R*. *solani* AG1-IA has been extensively studied due to its economic importance [11, 12]; however, studies examining its interactions with other economically important crops, such as soybean, have yet to be extensively studied. Recently, the draft genome sequence of AG1-IA rice isolate B275 was established and made publically available [12] enabling insights into the gene structure and functionality of the pathogen, and allowing for more comprehensive genetic studies of the AG1-IA rice pathogen. To date, only one study has utilized the genome to examine the global transcript responses of *R*. *solani* AG1-IA during root rot of turf grass (*Zoysia japonica*) [13].

Although certain effectors have been well studied in certain pathogens [14], studies examining the global omics fluctuations that occur following and during host invasion are few [15]. Little is known about the molecular components responsible for susceptibility or resistance of soybean to *R*. *solani* AG1-IA isolates. This information is crcuial because of the impact of RFB on agriculture. Understanding virulence of the pathogen and identifying genes during its interaction with soybean is not only undeniably vital for plant breeders, but would facilitate improved control strategies for this important pathogen.

Omics technologies allow for a more in-depth understanding of biological system responses to perturbations and are a promising tool for the examination of host-pathogen interactions. RNA sequencing (RNA-seq) provides a largely unbiased method to comprehensively and systematically define the transcript fluctuations of an organism in a manner that is significantly more sensitive than microarray hybridization approaches [16]. This technology has become available as a powerful tool to investigate the transcriptional profiles of microbes including plant pathogens [13, 17, 18]. RNA-seq is able to identify novel fungal pathogen transcripts such as pheromone receptors [12] and capsule formation genes [13], advocating its use as a promising tool for understanding plant-pathogen interactions, and in extent for development of alternative control methods and resistant cultivars.

**Fig 1:**
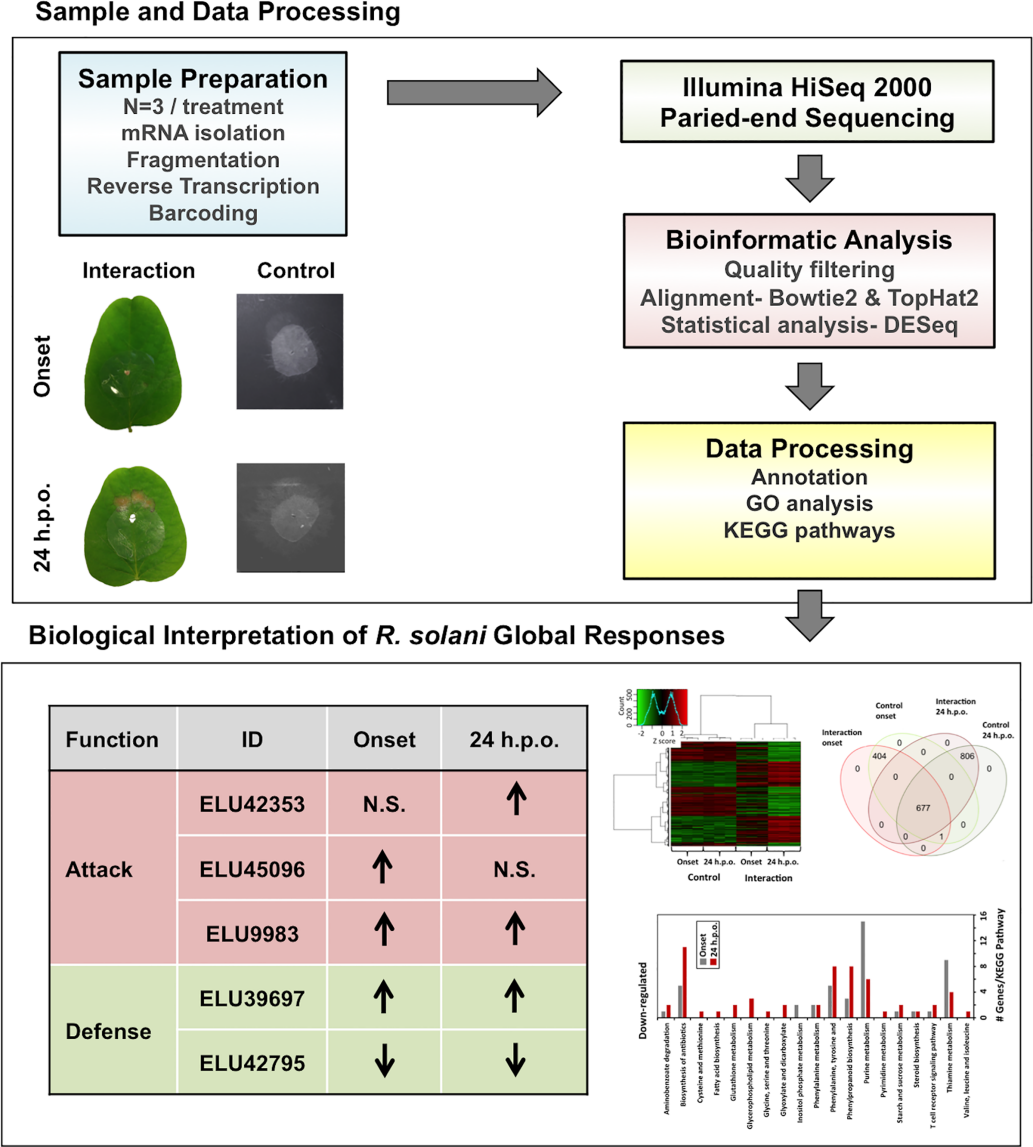
Flowchart of steps taken for *R*. *solani* AG1-IA RNA-seq sample preparation and data analysis. Hyphae of *R*. *solani*, grown on ¼ strength PDA (control) or on soybean unifoliate leaves were harvested and processed for RNA sequencing using the Illumina HiSeq 2000 platform. Data were analyzed using standard analytical pipelines for gene annotation and differential expression analysis. Data were further compared using heatmap analysis and gene ontology terms, and affected pathways examined using Kyoto encyclopedia of genes and genomes (KEGG).

Here, we have taken advantage of high throughput RNA-seq to report a first comprehensive study on global transcriptome fluctuations of *R*. *solani* AG1-IA strain ROS-2A4 at onset and late necrosis stages of RFB disease development on soybean. The detailed *in silico* analysis (**Fig 1**) revealed modulation of multiple molecular pathways, and fluctuations in genes encoding virulence factors and stress responses whose expression analysis reflected many that exhibit differential gene expression during disease development.

## Materials and methods

### Growth and maintenance of *Rhizoctonia solani* AG1-IA

The highly virulent *Rhizoctonia solani* AG1-IA strain ROS-2A4 was obtained from P. Ceresini, University of São Paulo State (UNESP), Brazil. *R*. *solani* cultures were revived from stock cultures maintained at -80°C by placing mycelial plugs on fresh potato dextrose agar (PDA) for one week at 24°C in the dark. One-week-old cultures were sub-cultured onto fresh PDA until sclerotia initials began forming (approximately 10 days). Sclerotia initials were used for *R*. *solani* inoculation experiments.

### Soybean growth and *Rhizoctonia solani* infection conditions

Soybean (*Glycine max*) cv. Williams 82 seeds were surface sterilized in 30% hydrogen peroxide for 7 minutes followed by 5 rinses in sterile water and imbibed on sterile filter paper moistened with 20 mL of water for 48 hours. Seeds with similar emergence rates were planted in Cone-tainers® containing 130 mL of Agro-Mix G10 (Fafard, Ltd., Saint-Bonaventure, Canada) and sand (1:1 v/v) and watered with 30 mL of sterile water every two days. To study *R*. *solani* disease progression, a detached leaf assay was conducted. These assays have been shown to have high correlations between field and greenhouse pathology trial assessments [19, 20]. To do so, 20 fully expanded soybean unifoliate leaves originating from 20 different plants were detached from 2 week-old plants. Leaves were then placed on a moistened filter paper in 15 cm Petri dishes such that each Petri dish contained 5 unifoliate leaves. A biological replicate consisted of 4 Petri dishes containing 20 pooled leaves. A total of 3 biological replicates were prepared for each time point.

**Fig 2:**
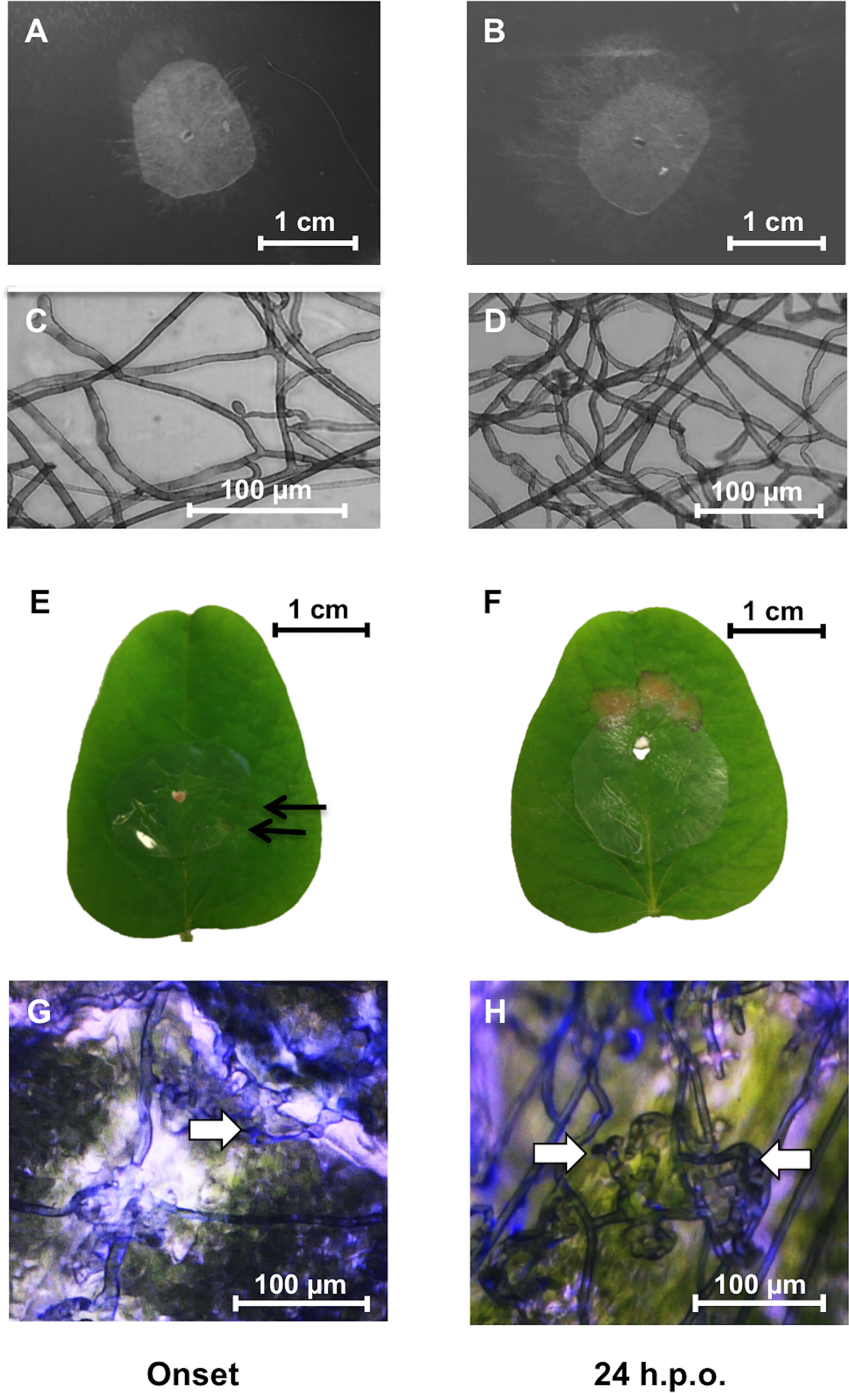
*Rhizoctonia solani*-soybean interactions at onset and 24 h.p.o. of necrosis. *In vitro* controls on PDA harvested at onset (**A**) and 24 h.p.o. (**B**) of necrosis. Microscope images of hyphae on nitrocellulose membranes *in vitro* at onset (**C**) and 24 h.p.o. (**D**) of necrosis showing normal growth and lack of hyphal aggregates and infection structures. Soybean leaf samples infected with *R*. *solani* AG1-IA at onset (**E**) and 24 h.p.o. (**F**) of necrosis. Arrows indicate the onset of necrotic lesions approximately 36 h post-inoculation. Microscope images of hyphae on nitrocellulose membranes from soybean leaves at onset (**G**) and 24 h.p.o. (**H**) of necrosis showing infection cushion structures (arrows). Note that hyphal aggregation and infection cushion initials occurred only in *R*. *solani* samples grown on membranes overlaid on leaves (**G, H**) and not on PDA (**C, D**).

In order to obtain the maximum number of RNA-seq reads from *R*. *solani*, an infection system was developed enabling the collection of sufficient mycelia for RNA-seq library construction with minimal soybean RNA contamination. A sterile moistened nitrocellulose membrane (1 cm in diameter) was placed in the center of each unifoliate soybean leaf, and inoculated with a sclerotia initial of *R*. *solani* AG1-IA placed in the middle of the cellulose membrane (**Fig 2**). Control treatments consisted of sclerotia initials placed in the center of cellulose membranes (1 cm diameter) overlaid on ¼ strength PDA. The use of membranes in interaction and control treatments facilitates the harvesting of the mycelium while excluding unwanted plant tissue or PDA, respectively. The use of porous nitrocellulose membranes is a common practice when studying *R*. *solani* molecules during plant [21, 22] and microbe-microbe interactions [23]. The absence of infection structures and hyphal aggregates on membranes from samples grown on PDA (**Fig 2**) corroborates earlier findings that these structures are not governed by contact stimuli [22, 24]. Petri dishes were sealed with parafilm and placed in growth chambers at 24/22°C day/night with 12 h day/night cycles and humidity maintained at 65% throughout the day. Samples were harvested at onset and 24 h post-onset (h.p.o.) of necrosis **(Fig 2).** Onset of necrosis was determined by examining the leaves every hour until necrotic lesions (<1 mm), hyphal aggregation and infection cushion initials were visible on the leaf and the overlaid membrane (**Fig 2G and 2H**). The mycelia from control samples (PDA) and infected leaves were peeled from the membranes and immediately flash frozen in liquid nitrogen until further use. A total of 20 membrane discs were pooled together for each sample for a total of 3 biological replicates per treatment per time point.

### RNA extraction and RNA-seq library preparation

Total RNA was extracted from mycelial samples using Trizol® (Diamed, Mississauga, Canada) following the manufacturer protocols. Briefly, 50 mg of hyphae per sample were ground to a fine powder in liquid nitrogen and 1 mL of Trizol added to each sample. Total RNA in the supernatant was purified using a chloroform wash, precipitated using isopropanol and dissolved in RNase-free water. Total RNA quantity and quality were measured using a NanoDrop ND-1000 Spectrophotometer (Thermo Scientific, Wilmington, DE, USA) and denaturing formaldehyde gel electrophoresis, respectively. RNA-seq libraries were prepared for each replicate from 4 μg total RNA using the KAPA stranded mRNA-Seq kit (KAPA Biosystems, Inc., Wilmington, MA) with slight modifications. Briefly, mRNA was captured and purified by performing two purifications on the KAPA mRNA capture beads by mixing total RNA with 50 μL of beads, heating at 65°C for 5 minutes and cooling with shaking at 150 rpm at room temperature for 20 minutes. cDNA libraries with fragment lengths of 200–300 bp mRNA were constructed following the manufacturer protocols. The 12 libraries were barcoded using NEXTflex RNA-seq barcodes (BiooScientific, Toronto, Canada) 1 through 12 (v1-1-15-1), and amplified using the following conditions: initial denaturation at 98°C for 45 s, followed by 12 cycles of denaturation at 98°C for 15 s, annealing at 60°C for 30 s and elongation at 72°C for 30 s, and a final extension at 72°C for 5 minutes. Libraries were purified using NucleoMag NGS Clean-up and Size Selection beads (Machery-Nagel, Bethlehem, PA, USA) and quantified by a NanoDrop ND-1000 Spectrophotometer, and the size and quality were confirmed by agarose gel electrophoresis (2%). Libraries were pooled such that one final library contained equal amounts of each of the 12 barcoded libraries and the final quality and quantity were confirmed using a bioanalyzer at the Genome Quebec Research Centre (Montreal, Canada). The library was sequenced at the Genome Quebec Research Centre (Montreal, Canada) and sequenced in one lane using the Illumina HiSeq 2000 system with 100 bp paired-end sequencing.

### Bioinformatics analysis

Illumina reads were separated by barcodes following the Genome Quebec pipeline (available at https://bitbucket.org/mugqic/mugqic_pipelines). Illumina adapters were removed and reads with average phred scores below 20 and lengths below 50 bp were removed using the software Trimmomatic version 0.35 [25]. Alignment of trimmed and clean reads was done for each library using TopHat2 v2.0.8b [26] with alignments to the *R*. *solani* AG1-IA genome and transcriptome Rhisol_AG1IA version 1.0 [12] available on NCBI (taxid: 983,506; BioProject Accession PRJNA51401), and also to the soybean genome v1.1 [27] available on the JGI Genome Portal (http://genome.jgi-psf.org/) using default settings. Reads aligning to the soybean genome were removed prior to alignment to the *R*. *solani* genome and annotation.

### Transcript relative quantification and differential abundance analysis

Reads were counted using HTseq version 0.5.4p3 [28] and normalized using the estimateSizeFactors and variance stabilized using varianceStabilizingTransformation commands in the R statistical package DESeq version 1.14.0 [29]. First, differences between time points and detection of outliers were detected using PCA and *Hotelling’s T*^*2*^ confidence (95%) analysis on normalized and variance stabilized sequence counts using SIMCA-P+ v.13.0.3.0 software (Umetrics). Statistical analysis for differential gene expression was performed using the R software package DESeq version 1.14.0 using negative binomial comparisons between all treatments [29]. Differentially expressed genes (DEGs) were identified using the following criteria: 1) a fold change value >3 or <-3 and; 2) a Benjamini-Hochberg [30] false discovery rate (FDR) <0.01. Heatmap analysis using variance stabilized data of expressed genes and the command heatmap.2 from the R package gplots [31]. A Venn diagram to compare the differentially expressed genes within each treatment was constructed using the R packages VennDiagram [32] and limma [33].

### Transcript annotation, functional annotation and pathway mapping

Sequence identification was done first by comparing reads to the publically available *R*. *solani* AG1-IA annotation fasta file available on the EnsemblFungi server (http://fungi.ensembl.org), and by using the BLASTx algorithm of the Blast2GO software version 3.2 [34, 35] at a statistical significance threshold of 1.0E-6. Functional categories were assigned using the Gene Ontology (GO) Slim terms using the Blast2GO software version 3.2 [34, 35]. Integration of InterPro Scan and ANNEX functions was done for improved functional annotation [34, 36, 37]. To determine if particular GO terms were over- or under-represented in the DEGs, enrichment analysis of the GO terms in all GO categories (biological process, molecular function and cellular component) was performed using a Fisher’s exact test and a FDR threshold <0.05. To determine to which metabolic pathways DEGs belonged, genes were assigned Kyoto Expedia of Genes and Genomes (KEGG) enzyme codes [38] using the Blast2GO GO-enzyme code mapping function [34] (**S1 and S2 Tables**).

### Quantitative real-time PCR validation

Differentially expressed genes identified from sequencing data were confirmed by qRT-PCR analyses on 13 genes (**S3 Table**). To do so, samples were harvested from a second trial conducted under identical conditions and similar time points as previously described. Total RNA was extracted from the second trial samples using the Trizol method described above and 1 μg was converted to cDNA using the QuantiText Reverse Transcription Kit (Qiagen, Toronto, Canada) following the manufacturer protocols. Each 15 uL qRT-PCR reaction contained 1X SsoAdvanced Universal SYBR Green Supermix (Bio-Rad Laboratories Ltd.), 0.175–0.25 μM each primer (**S3 Table)**, and 500 ng cDNA. The thermocycling profile used an initial denaturation at 95°C for 3 min, followed by 35 or 40 cycles of denaturation at 95°C for 30 s, annealing for 30 s at the appropriate primer temperature (**S3 Table**) and extension at 72°C for 40 s, followed by a dissociation curve analysis. Gene expression was analyzed using the method of [39] with normalization over the housekeeping gene histone 3 (ELU43810). Transcript levels were quantified in three biological replicates per treatment and significant differences determined using analysis of variance (ANOVA) and student’s *t* test at the 95% significance level using R statistics software. Fold change differences ≤ -1.5 or ≥ 1.5 with *P*<0.05 were used for qRT-PCR rather than ≤ -3 or ≥ 3 due to the differences in methods of normalization between RNA-seq and qRT-PCR [40, 41].

### Availability of supporting data

All data discussed in this publication have been deposited in the NCBI Sequence Read Archive (SRA) under the BioProject PRJNA369092.

## Results

### Transcriptomic analysis

**Fig 3:**
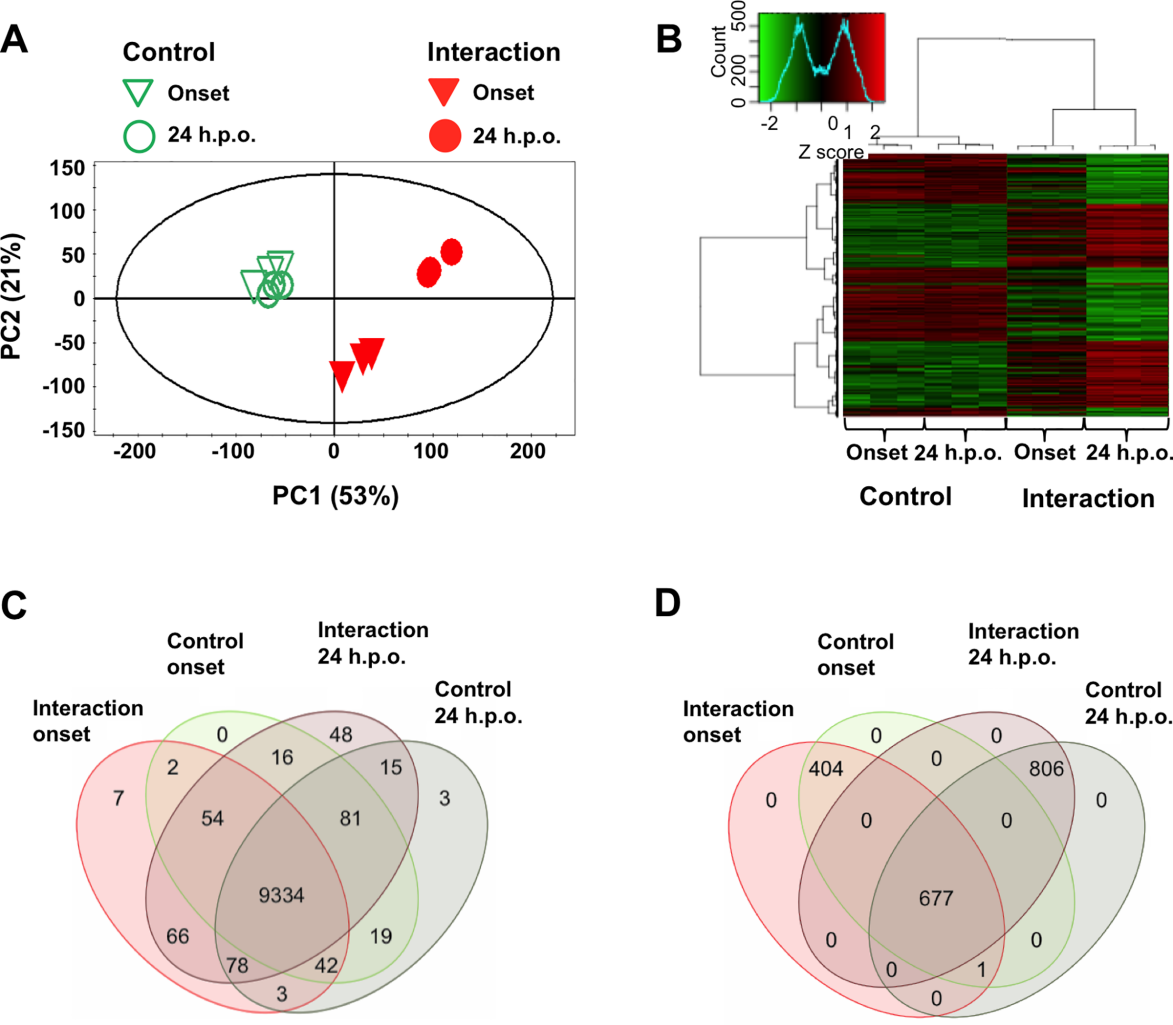
Overview of analysis of *Rhizoctonia solani* differentially expressed genes at onset and 24 h.p.o. of necrosis in soybean. (**A**) PCA score plot of *R*. *solani*-soybean interactions (solid inverted triangles and circles) and controls (open inverted triangles and circles) at onset of necrosis (inverted triangles) and 24 h.p.o. of necrosis (circles). (**B**) Heatmap and hierarchical cluster analysis of *R*. *solani*-soybean interactions at onset and 24 h.p.o. of necrosis. Dendrograms were constructed using the Ward method [42]. (**C**) Venn diagram of transcriptionally active genes (at least 2 reads per sample in 2/3 biological replicates for any treatment) and the treatments in which they were detected (*n* = 3 per treatment). (**D**) Venn diagram of the 1,888 differentially expressed genes and the treatments in which they were detected (*n* = 3 per treatment). A number of zero indicates that no genes were found to be unique to the specific comparison.

Onset of necrosis occurred approximately 36 hours post-inoculation (**Fig 2**), whereas full-blown necrosis was evident by approximately 60 hours post-inoculation (**Fig 2**). Libraries of twelve samples were sequenced on a single Illumina HiSeq 2000 sequencing lane resulting in high quality reads ranging from 11 to 13 million reads per sample (**S4 Table**). Alignment with TopHat2 showed that less than 1% of reads aligned to the soybean genome, indicating that the membrane method developed for leaf infection was effective and sufficient for extracting highly purified *R*. *solani* RNA during biological interactions. Principal component analysis (PCA) revealed tight clustering of all control samples and no outliers, irrespective of time of harvest, and clear separation of the soybean-*R*. *solani* interaction samples (**Fig 3A**). This was further supported by hierarchical cluster analysis (HCA) and heatmap analysis of the top 40% differentially expressed genes (**Fig 3B**). A clear separation was seen between fungal control and fungal interaction samples, with strong differential gene expression at 24 h.p.o. of necrosis, whereas DEG of fungal control samples were grouped together (**Fig 3A and 3B**). The differential expression data of onset of necrosis were grouped closer with those of both the control samples indicating that differential gene regulation was just commencing (**Fig 3A and 3B**).

Genes were considered transcriptionally active if there were at least two reads per sample in two out of three biological replicates of any of the treatments. A total of 9768 genes (93.1%) out of the 10,489 currently identified *R*. *solani* AG1-IA coding sequences were transcriptionally active across the two time points (**Fig 3C**) [12]. A total of 11% and 15% of the expressed transcriptome was differentially expressed representing a total of 1082 and 1484 differentially expressed genes (DEGs) with fold-change values of +/- 3 at onset of necrosis and 24 h.p.o. of necrosis compared to their controls, respectively (**S5 and S6 Tables**). Of the DEGs, 678 were common between the two time points (**Fig 3D**). Comparison between the two time points in infected leaves resulted in 727 DEGs (**S7 Table**).

### Annotation and gene ontology analysis of differentially expressed transcripts

Transcripts were annotated using the publically available annotated fasta file and BLASTx, followed by annotation enhancement using the InterproScan database [37] and the ANNEX augmentation procedure [36], resulting in a total of 1204 annotated genes or 63.8% of the DEGs. The top 20 annotated differentially up- and down-regulated genes at each time point are presented in **S8 and S9 Tables** respectively.

Enrichment analysis of the DEGs with the reference genome revealed significantly under- and over-represented gene ontology (GO) slim terms [43] at both time points (**Table 1**). Fewer GO slim terms were affected at onset compared to 24 h.p.o. of necrosis, with GO terms being under-represented at onset of necrosis. A total of 3 GO slim terms were under-represented at onset, whereas no over-representation was observed at this time point. Four under-represented and 23 over-represented GO slims were identified 24 h.p.o. (**Table 1**). Of these, none were common between the two time points.

**Table 1:** Gene ontology slim terms over- and under-represented in *Rhizoctonia solani* during soybean infection.^a^

	GO Slim ID	Term	Classification^b^
**Onset, Under-represented**	GO:0005623	cell	C
	GO:0044464	cell part	C
	GO:0005622	intracellular	C
**24 h.p.o., Over-represented**	GO:1990904	ribonucleoprotein complex	C
	GO:0030529	intracellular ribonucleoprotein complex	C
	GO:0005840	ribosome	C
	GO:0043228	non-membrane-bounded organelle	C
	GO:0043232	intracellular non-membrane-bounded organelle	C
	GO:0003735	structural constituent of ribosome	F
	GO:0005198	structural molecule activity	F
	GO:0006518	peptide metabolic process	P
	GO:0006412	translation	P
	GO:1901566	organonitrogen compound biosynthetic process	P
	GO:0034645	cellular macromolecule biosynthetic process	P
	GO:1901576	organic substance biosynthetic process	P
	GO:0044249	cellular biosynthetic process	P
	GO:0043043	peptide biosynthetic process	P
	GO:0043604	amide biosynthetic process	P
	GO:0043603	cellular amide metabolic process	P
	GO:0009059	macromolecule biosynthetic process	P
	GO:0044271	cellular nitrogen compound biosynthetic process	P
	GO:1901564	organonitrogen compound metabolic process	P
	GO:0042254	ribosome biogenesis	P
	GO:0010467	gene expression	P
	GO:0022613	ribonucleoprotein complex biogenesis	P
	GO:0044085	cellular component biogenesis	P
**24 h.p.o., Under-represented**	GO:0043227	membrane-bounded organelle	C
	GO:0043231	intracellular membrane-bounded organelle	C
	GO:0005634	nucleus	C
	GO:0016740	transferase activity	F

^a^Enrichment analysis of the GO terms in all GO categories (biological process, molecular function and cellular component) was performed using a Fisher’s exact test and an false discovery rate threshold <0.05.

^b^Classification based on cellular component (C), molecular function (F) or biological process (P).

### Metabolic classification of *Rhizoctonia solani* differentially expressed genes

**Fig 4:**
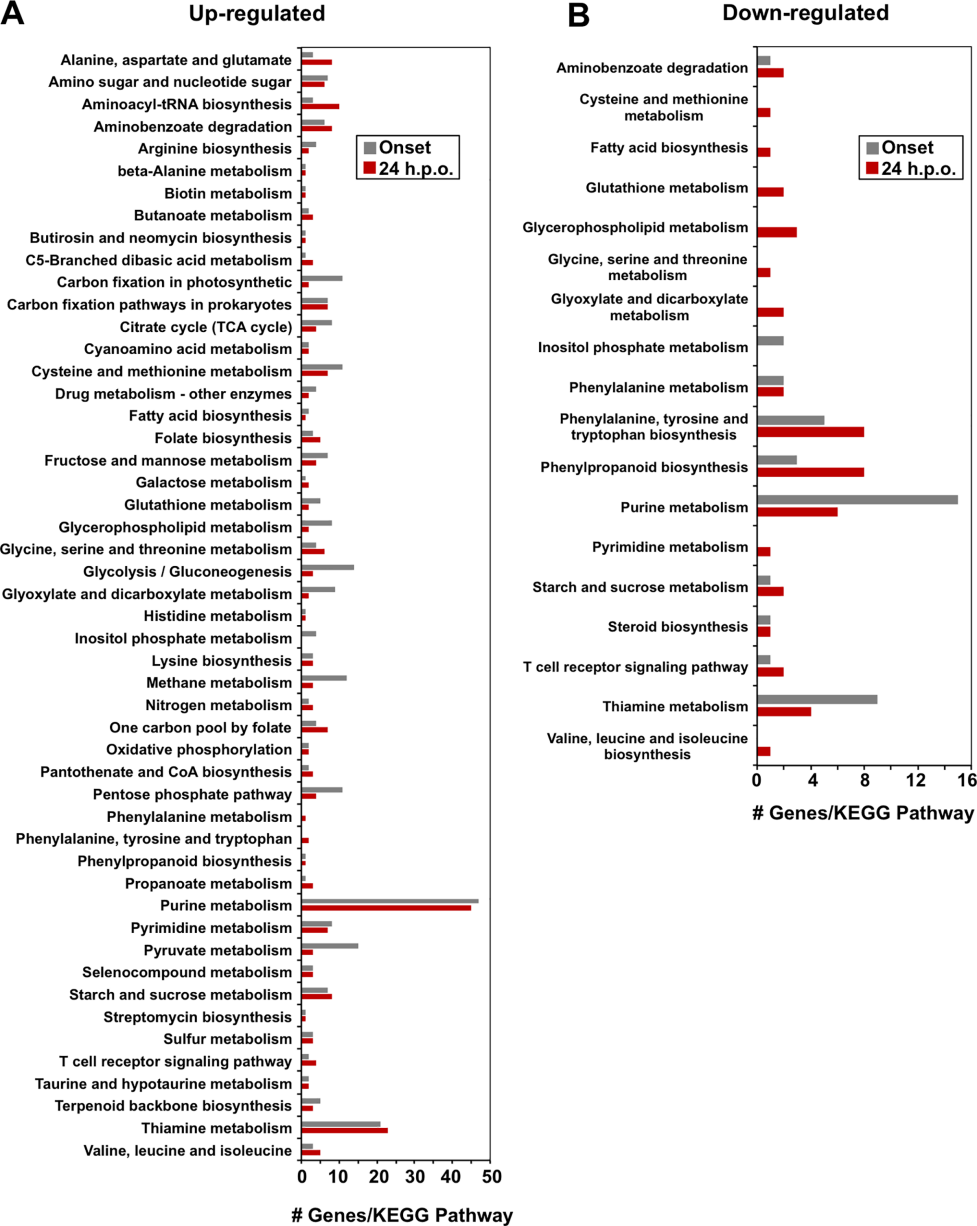
KEGG pathway annotations common between *Rhizoctonia solani*-soybean interaction time points. KEGG pathway annotations common between *R*. *solani*-soybean interactions at onset (grey) and 24 hours-post onset (red) of necrosis for differentially expressed genes. (**A**) KEGG pathways commonly containing up-regulated DEGs, (**B**) KEGG pathways commonly containing down-regulated DEGs (*n* = 3 per treatment). Numbers represent the number of differentially expressed genes with fold change values +/- 3 detected for each KEGG pathway.

KEGG pathway analysis indicated that DEGs were implicated in a number of pathways during *R*. *solani-*soybean interactions (**Fig 4 and S1 Fig**). Mapping of annotated DEGs to KEGG pathways resulted in 116 DEGs mapping to 61 pathways and 151 DEGs mapping to 64 pathways at onset and 24 h.p.o. of necrosis, respectively, for a total of 52 KEGG pathways containing DEGs affected at both time points **(Fig 4).** The highest representation among genes that were up-regulated vs down-regulated at onset and 24 h.p.o. of necrosis were involved in purine metabolism (47 and 45 genes vs. 15 and 6, respectively) and thiamine metabolism (21 and 23 vs 9 and 4, respectively).

In some cases, very few DEGs mapped to particular KEGG pathways, including those that are up-regulated and associated to toxin degradation (nicotinate and nicotinamid metabolism), the synthesis of anti-microbial compounds (aflatoxin biosynthesis, novobiocin biosynthesis, and penicillin and cephalosporin biosynthesis) and pyridoxine (vitamin B6) biosynthesis at either onset or 24 h.p.o of necrosis (**S1 Fig**). Similarly, very few down-regulated genes were mapped to KEGG pathways involved in the degradation of xenobiotics as well as ascorbate and aldarate metabolism (**S1 Fig**).

### Validation of transcripts by real-time PCR

**Fig 5:**
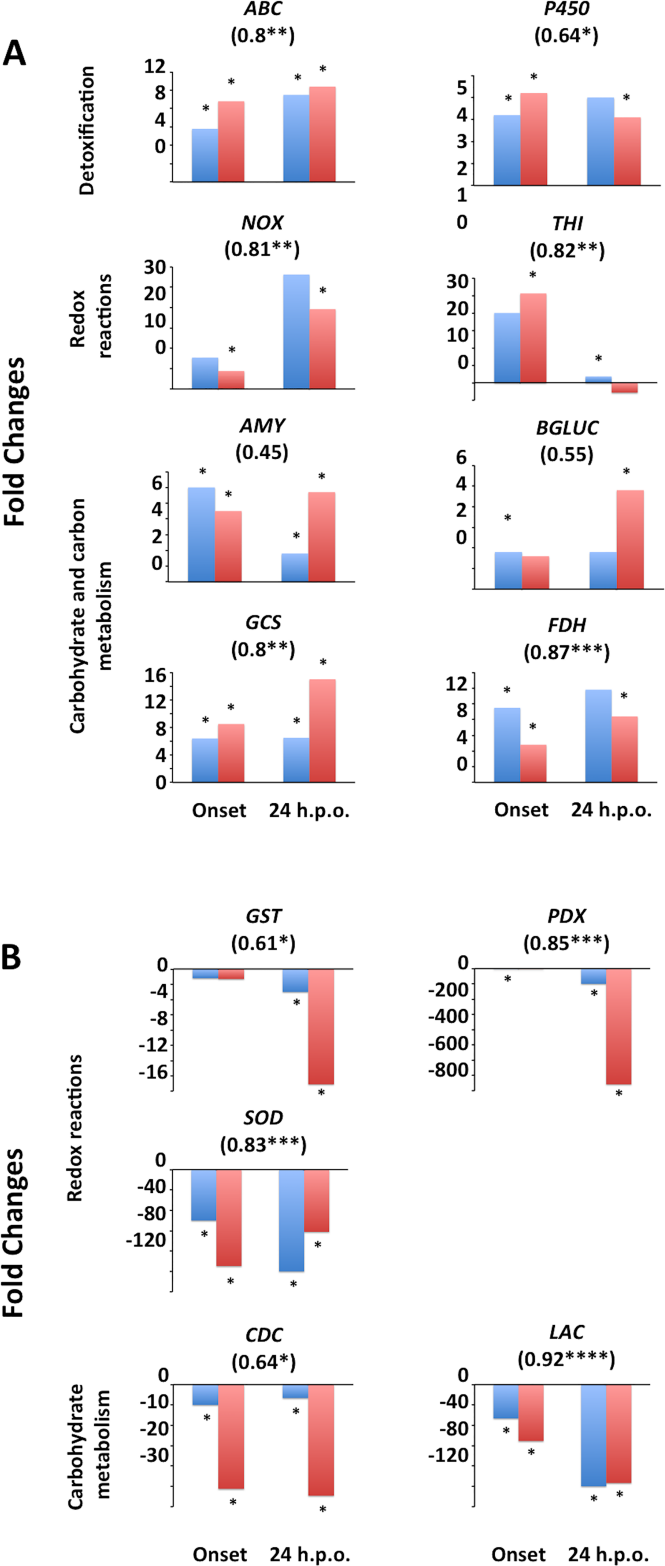
*Rhizoctonia solani* transcript abundance fold changes in response to infection of soybean. Transcript abundances were quantified using qRT-PCR (blue) or RNA-seq (red) in *R*. *solani* cultures infecting soybean compared to controls *in vitro* at onset of necrosis or 24 h.p.o. of necrosis. (**A**) Up-regulation of transcripts during infection of soybean. (**B**) Down-regulation of transcripts during infection of soybean. Stars represent fold changes that were statistically (*P*<0.01 for RNA-seq or *P*<0.05 for qRT-PCR) and biologically (fold change ≤ -3 or ≥ 3 for RNA-seq or ≤ -1.5 or ≥ 1.5 for qRT-PCR) significant (*n* = 3 per treatment). Numbers below gene names represent Spearman’s correlation coefficients with stars representing significance thresholds of: *P ≤0.05, **P ≤0.01, ***P ≤0.001 or ****P ≤0.0001. *ABC*, ABC transporter; *AMY*, *ALPHA-AMYLASE*; *BGLUC*, *BETA-GLUCOSIDASE*; *CDC*, *CHITIN DEACETYLASE*; *FDH*, *FORMATE DEHYDROGENASE*; *GCS*, *GLYCOGEN SYNTHASE*; *GST*, *GLUTATHIONE-S-TRANSFERASE*; *LAC*, *LACCASE PRECURSOR*; *NOX*, *NADH OXIDASE*; *P450*, *CYTOCHROME P450 MONOXYGENASE PC-3*; *PDX*, *PYRIDOXAL-DEPENDENT DECARBOXYLASE*; *SOD*, *Cu/Zn SUPEROXIDE DISMUTASE*; *THI*, *THIAMINE BIOSYNTHESIS*.

Thirteen genes, including 5 (*NOX*, *THI*, *GST*, *PDX*, *SOD*) linked with oxidative stress function, 6 (*CDC*, *LAC*, *AMY*, *BGLUC*, *GCS*, *FDH*) with carbohydrate and carbon metabolism, and 2 (*ABC*, *P450*) with detoxification or degradation of toxins were validated to be differentially expressed at the two time points using qRT-PCR (**Fig 5**). Examination of transcript abundance between RNA-seq and qRT-PCR transcript abundances showed similar trends between the two methods (**Fig 5**). Correlation analysis was performed between RNA-seq and qRT-PCR relative abundances for each gene. Among the 13 genes, 8 (*ABC*, *NOX*, *THI*, *GCS*, *FDH*, *PDX*, *SOD* and *LAC*) were highly correlated (*r* ≥0.75), 3 (*P450*, *GST* and *CDC*) were moderately correlated (*r* ≥0.5 and <0.75) and 2 (*AMY* and *BGLUC*) had poor correlations (*r* <0.5) (*P<*0.05). Although variations in correlations were observed as samples originated from different trials, and the two methods use different calculations for normalization of transcripts, strong correlations and similar trends in transcript abundances were generally observed confirming the observed trends in changes in transcript abundances.

## Discussion

The use of RNA-seq has provided substantial insights into plant-pathogen interactions; however, only recently have studies begun to examine the global transcriptome responses of phytopathogens such as *Rhizoctonia solani* while infecting host plants [12, 13]. Although RNA-seq can provide information on both the host and pathogen, previous studies have reported that such studies provide few reads and therefore little information on the pathogen responses [41, 44, 45]. As such, a method using nitrocellulose membranes to obtain an in-depth and thus highly informative study of *R*. *solani* responses during soybean infection was developed, similar to studies examining *R*. *solani* during plant [21, 22] and microbe-microbe [23] interactions. This method allowed for the vast majority of sequences to belong to *R*. *solani*, rather than its soybean host. Still, however, a large amount of reads (±30%) did not align to the *R*. *solani* reference genome. We speculate that this may be the result of: sequencing errors, quality thresholds for passing alignments, an incomplete reference genome are all legitimate reasons that could explain the low alignment. Additionally, the transcriptome used for alignment belonged to a divergent *R*. *solani* AG1-IA strain than that used in our study resulting in strain specific transcripts or genomic regions. Similar results have been reported for other phytopathogen RNA-seq studies [45].

The results presented in this study demonstrate that when *R*. *solani* infects soybean leaves, it regulates expression of genes associated with defence and attack. Functional annotation of these genes based on GO terms revealed that several of them encode important cellular, molecular and biological functions involved in defence through the synthesis of antioxidants for ROS quenching, manipulation of the surrounding environment, and attack via the synthesis of toxins, cell wall degrading enzymes and the use of alternative carbon sources. Significant differences in genes involved in certain KEGG metabolic pathways and individual genes involved in attack and defence during early (i.e., onset of necrosis) and late infection (i.e., 24 h.p.o. of necrosis) stages of *R*. *solani* were evident.

### Defense

Necrotrophs such as *R*. *solani* require that the tissue be dead and externally digested prior to utilizing the plant nutrients. This destructive form of nutrient acquisition results in aggressive plant defense and attack mechanisms to limit the damage done by the pathogen. Our results indicate that *R*. *solani* employed two mechanisms to combat against soybean defenses, 1) ROS quenching, and 2) manipulation of intra- and extra-cellular environments.

#### 1) ROS quenching

Synthesis of reactive oxygen species (ROS) generally occurs during the initial defense responses of plants acting as both defense compounds and signalling molecules [46]. As such, antioxidant synthesis in phytopathogens is commonly observed throughout the infection process. Fluctuations in *Rhizoctonia* genes resulting in synthesis of antioxidants and ROS quenching proteins were observed at onset and 24 h.p.o. of necrosis during *R*. *solani-*soybean interactions (**S5 and S6 Tables**), with decreases at 24 h.p.o. compared to onset (**S7 Table**). *OXIDOREDUCTASE* and *OXIDASE* genes provide means of inhibiting or diminishing the potency of ROS by converting them to less reactive forms. At early infection stages, *R*. *solani* penetrates its host cells and must defend itself against the sudden increase in ROS during the plant hypersensitive response (HR) [46]. The likelihood that protection of *R*. *solani* cells against ROS damage is reduced or stopped by the action of antioxidants was established by the up-regulation of 5 *OXIDOREDUCTASE* and 10 *OXIDASE* genes at onset of necrosis, including 3 *NADH OXIDASE* encoding genes, 2 *GMC OXIDOREDUCTASE* genes, and a gene encoding *GLUTATHIONE-S-TRANSFERASE*. Fold changes of these genes ranged from 3.1 to over 32.9 during infection (**S5 and S6 Tables**) and transcript abundances were significantly higher at onset compared to 24 h.p.o. of necrosis (**S7 Table**). Congruent with these results is the induction of transcripts related to antioxidants in *R*. *solani* when challenged with antagonistic bacteria *Serratia proteamaculans* and *S*. *plymuthica* [47], and with the mycoparasite *Stachybotrys elegans* [23], or during *R*. *solani-*wheat interactions [21, 48]. During late infection of soybean leaves, however, a general trend of down-regulation of *OXIDOREDUCTASE* and *OXIDASE* genes, as well as *GLUTATHIONE-S-TRANSFERASE* was observed suggesting that at this stage the plant had most likely succumbed to the pathogen as evidenced by the high levels of necrosis.

Genes related to several antioxidant pathways, such as thiamine (vitamin B1), riboflavin (vitamin B2), ascorbate (vitamin C) and pyridoxine (vitamin B6) were induced 3.9 to 25.6 fold at onset of necrosis, whereas only genes involved in thiamine and pyridoxine biosynthesis were up-regulated 24 h.p.o. of necrosis with fold changes of 4.6 and 3.3, respectively (**S5 and S6 Tables**). When comparing fold changes between onset and 24 h.p.o., genes involved in thiamine and pyridoxine biosynthesis were up-regulated 17.3 and 162.5 fold, respectively (**S7 Table**). Pyridoxine and thiamine biosynthetic genes are known to act as antioxidants to relieve ROS stress during abiotic and biotic stress in fungi. It has been shown that thiamine biosynthesis is down-regulated when *R*. *solani* is challenged with the mycoparasite *Stachybotrys elegans* [23], while pyridoxine biosynthesis pathway was induced during abiotic stresses [49] suggesting that these pathways have roles in relieving oxidative stress in *R*. *solani*. This is the first report of the involvement of thiamine and pyridoxine biosynthetic genes during interaction of *R*. *solani* with its host.

Although the induction of gene expression holds true for the majority of genes involved in ROS quenching and antioxidant synthesis, a few notable exceptions were observed. Two genes encoding *COPPER-ZINC SUPEROXIDE DISMUTASE* (*SOD*) (ELU42795 and ELU42796; **S5 and S6 Tables**) were down-regulated at both time points with fold changes ranging from 0.1 to 0.17, a finding similar to that reported in *R*. *solani* during invasion of wheat [48], suggesting that antioxidants may have highly specific roles during particular interactions and/or stages of interactions.

#### 2) Manipulation of intra- and extra-cellular environment

Some fungi are capable of altering their environment to favour their survival by secreting enzymes such as *OXALATE DECARBOXYLASE* [50, 51]. High levels of oxalate in advance of a developing fungus may render plant tissue more susceptible to invasion as a result of calcium precipitation from the middle lamella of cell walls [52, 53]. In our study, two *OXALATE DECARBOXYLASE* genes of *R*. *solani* were up-regulated at both time points (**S5 and S6 Tables**), with similar amounts between the two time points. Similar overexpression of oxalate decarboxylase was also observed when an *R*. *solani* strain belonging to AG3 was challenged with two species of *Serratia* bacteria [47] and when rice was infected with a strain of *R*. *solani* AG1-IA [54]. The reason for the up-regulation is not clear, but it may imply that *R*. *solani* attempts to recycle the remaining oxalate, or that intra- or extra-cellular oxalate levels are high enough to pose a threat to the pathogen, a notion that remains open for speculation.

During host-pathogen interactions toxin synthesis by both partners is unavoidable. To minimize the intracellular concentration of toxins and secondary metabolites produced during pathogenesis, phytopathogens must be able to export these compounds from the target sites. Several genes encoding transporters, including several *ABC TRANSPORTER*, *MULTIDRUG TRANSPORTER* and *CHROMATE ION TRANSPORTER*, were differentially expressed during *R*. *solani* interactions with soybean (**S5 and S6 Tables**) suggesting a putative role in the protection of *R*. *solani* from soybean metabolites. Up-regulation of *ABC TRANSPORTER* has also been reported for *R*. *solani* in the presence of *Serratia* bacteria [47], and during invasion of the lawn grass *Zoysia japonica* [13] and rice [54].

The pigment, melanin is required to protect fungi and enhance their survival during adverse conditions, and it is also involved in sclerotial development [55]. One possible way for fungi to yield melanin is by catalyzing the oxidation of tyrosine via the action of tyrosinases [56]. In our study, we have identified 5 putative *TYROSINASE* genes that were differentially expressed at both time points (**S5 and S6 Tables**) and between time points (**S7 Table**) during infection of soybean leaves by *R*. *solani* AG1-IA. Consistent with this result, the EST dataset of *R*. *solani* AG1-IB (isolate 7/3/14), another subgroup of AG1 causing bottom rot of lettuce, also contained two *TYROSINASE* genes during exposure to lettuce exudates [57]. Generally, melanin is not known to have a direct role in fungal pathogens; however, some phytopathogenic fungi produce melanized infective structures for efficient pathogenicity [58]. The fact that 4 out of 5 *R*. *solani TYROSINASE* genes were down-regulated in our isolate at post-necrotic stages of soybean leaves suggests that tyrosinases may have a role during host penetration although this warrants further investigation.

Some members of the phylum Basidiomycota produce a polysaccharide capsule, comprised of xylose, mannose and gluconic acid, that protect them against environmental perturbations and are also reported to be involved in fungal virulence [59]. The EST dataset of *R*. *solani* subgroup AG1-IB includes sequences of key enzymes necessary for synthesis of a polysaccharide capsule: *PHOSPHOMANNOSE ISOMERASE* (*PMI*), *PHOSPHOMANNOMUTASE* (*PMM*) and *GDP MANNOSE PYROPHOSPHORYLASE* (*GMPP*) [57]. Formation of a polysaccharide capsule may protect the fungus against plant chitin degrading enzymes such as 1,3-beta-glucosidases. In our study, RNA-seq data of *R*. *solani* AG1-IA revealed the overexpression of a gene encoding *PMI* (ELU39697) at onset and 24 h.p.o. of necrosis (**S5 and S6 Tables**), with no differences between time points, suggesting an increased defense response to strengthen the cell wall and diminish the effects of plant chitin-degrading enzymes. Another putative polysaccharide capsule formation gene, *GDP-MANNOSE 4*,*6-DEHYDRATASE* gene (*GMD*; ELU42839), which has an important role in virulence of human pathogenic bacteria [60], was substantially over-expressed in *R*. *solani* 24 h.p.o. of necrosis, supporting the theory that polysaccharide capsules may have a role in *R*. *solani* virulence and defense. Interestingly, increases of polysaccharide capsule formation genes coincided with decreases in expression of *CHITIN SYNTHASE D* (*CSD*) and 3 *CHITIN DEACETYLASE* (*CDC*) genes at both time points compared to *in vitro* controls (**S5 and S6 Tables**), although higher *CDC* was observed at 24 h.p.o. compared to onset in samples infecting soybean (**S7 Table**). Acting primarily at hyphal tips, *CSD* aids in elongation of hyphae, while *CDC* converts chitin to chitosan making the hyphae more flexible for growth [61]. Reductions in these genes suggest that hyphal growth was decreased in *R*. *solani* during interaction with soybean compared to that growing on PDA, but was higher 24 h.p.o. compared to onset from soybean infecting samples. This, in conjunction with up-regulation of some polysaccharide capsule forming genes, strongly implies that *R*. *solani* has the ability to restructure its cell walls in order to protect itself from plant secondary compounds and fungal cell wall degrading enzymes. Similar results were observed when *R*. *solani* was challenged with species of the antagonistic bacteria *Serratia* [47].

### Attack

Necrotrophic fungi must not only overcome plant defenses to survive, but must have innovative attack mechanisms that will kill their host but not themselves. Large fluctuations of attack responses were observed in *R*. *solani* AG1-IA during soybean infection, including: 1) toxin synthesis; 2) synthesis of plant cell wall and carbohydrate degrading enzymes; and 3) use of alternative carbon sources.

#### 1) Toxin synthesis

Several ricin-type beta-trefoil lectin domain-containing proteins were temporally affected throughout the study (**S5 and S6 Tables**) with higher expression levels at onset of necrosis compared to 24 hp.o. (**S7 Table**). *Rhizoctonia solani* lectins have been associated with insecticidal activity [62], carbohydrate storage [63], fungi-fungi interactions [64] and cell surface and extracellular environment recognition [64]. Up-regulation of *RICIN-TYPE BETA-TREFOIL LECTIN* genes and proteins was observed during *R*. *solani* AG3 confrontation with *Serratia* bacteria [47] and confrontation of *R*. *solani* AG8 with wheat [21], respectively, suggesting an important role during biotic stress and plant invasion (this study). Interestingly, ricin-like lectins are involved in the production of the legume-specific toxin ricin and the up-regulation of ricin-like lectins observed here and in other studies [21, 47] suggest that they may have a role in *R*. *solani* toxin production; however the ricin-A domain, which is required for proper ricin protein function in plants, was not observed. Taken together, these results suggest that these domain-containing genes merit investigation for their role in *R*. *solani* adhesion, successful host penetration, attack and defense, and growth to determine the exact role of the ricin-B domain in *R*. *solani*.

The RNA-seq transcripts analysed in this study comprised of different genes involved in secondary metabolite biosynthesis, such as *VELVET* genes. The velvet (VeA) family of fungal regulatory proteins is linked to coordination of secondary metabolism and development [65]. In *Aspergillus nidulans*, mutants lacking the ability to produce VELVET proteins are incapable of producing the aflatoxin precursor sterigmatocystin and cannot produce fruiting bodies [66]. VELVET proteins also appear to have roles in *Trichoderma virens* secondary metabolite synthesis and mycoparasitism of *R*. *solani* [67], and isotigs featuring homology to aflatoxins have been identified in the *R*. *solani* AG1-IB genome [57]. In our study, a *VELVET DOMAIN-CONTAINING* gene was up-regulated 24 h.p.o. of necrosis compared to *in vitro* controls and onset of necrosis in samples infecting soybean (**S6 and S7 Tables**). Generally, these results suggest a variety of roles for these proteins in toxin and secondary metabolite synthesis, and possibly during plant invasion.

#### 2) Synthesis of plant cell wall and carbohydrate degrading enzymes

Phytopathogens produce plant cell wall degrading enzymes that are essential for host penetration and invasion. *R*. *solani* AG1-IA has an expanded set of putative genes encoding cell wall degrading enzymes (e.g., pectinases, cellulases and ligninases). Some, such as those encoding *PECTATE LYASE (PL*) and *CARBOHYDRATE ESTASE* (*CE*), were highly expressed at onset of necrosis (**S5 Table**), while others related to the degradation of cutin, cellulose, and carbohydrates were up-regulated 24 h.p.o. of necrosis (**S6 Table**). Currently, 29 fungal glycoside hydrolase (GH) families are known to be involved in plant biomass degradation [68]. In our study, 18 genes encoding *GH*s were differentially expressed during *R*. *solani-*soybean interactions (**S5 and S6 Tables**), with some up- or down-regulated at both time points, and others at only one time point. Of these, one gene (ELU41238) belonged to GH family 35, which is known to be involved in plant cell wall degradation [68], was down-regulated and unaffected at onset and 24 h.p.o. of necrosis, respectively. Similar expression patterns have been reported during the interaction of different anastomosis groups of *R*. *solani* with their respective hosts including wheat [21], rice [12] and turf grass [13], as well as when grown on media amended with host-derived root exudates [57]. Collectively, these results indicate the existence of a universal mechanism underlying plant cell wall degradation by phytopathogenic fungi. The remaining GHs belonged either to GH families involved in fungal cell wall degradation (typically down-regulated at both time points) or energy storage and recovery (typically up-regulated at both time points) [68]. Interestingly, when comparing the two time points of samples infecting soybean, higher levels of GH abundances were observed 24 h.p.o. compared to onset of necrosis (**S7 Table**). This, along with the previously discussed expression patterns of *CHITIN DEACETYLASE* and *CHITIN SYNTHASE*, suggests a general growth and excess of energy available to the fungus grown on plants compared to when grown *in vitro*.

Laccase enzymes are key enzymes involved in lignin degradation and two *LACCASE* genes, belonging to the multicopper oxidase group, were differentially expressed throughout the study: one *LACCASE* (*LAC*) was up-regulated at onset of necrosis (**S5 Table**) and the other was down-regulated 24 h.p.o. of necrosis (**S6 Table**), and up-regulated at onset compared to 24 h.p.o. (**S7 Table**). Laccases oxidize molecules, such as lignin, phenols and aromatic amines, although fungal laccases appear to be substrate non-specific [69]. Despite their potential diverse roles, it is generally found that fungal laccases are activated in high carbon to nitrogen environments or during low sugar (glucose or sucrose) scenarios [69]. *LACCASE* gene expression is commonly reported as up-regulated during stress as in the case of interaction of different *R*. *solani* AGs with *Serratia* species [47], lettuce exudates [57], and potato and lupin [70]. Therefore, the up-regulation of *R*. *solani LACCASE* reported here, suggests that there is a lack of readily available nutrients or high stress at onset of necrosis, while its down-regulation at the later stage may signify an excess of readily available nutrients and/or reduced stress due to the high level of plant necrosis.

#### 3) Use of alternative carbon sources

During attack, phytopathogens must be capable of responding to a rapidly changing environment in order to survive. One such strategy is their ability to utilize alternative carbon sources for energy. Two key enzymes in the glyoxylate pathway, *ISOCITRATE LYASE* (*ICL*) and *MALATE SYNTHASE* (*MLS*), aid in host penetration and disease development of several plant pathogenic fungi by relying on the catabolic products of lipids such as fatty acids and of carboxylic acids such as acetate as energy sources [71–74]. The up-regulation of *ICL* and *MLS* at onset of necrosis (**S5 and S7 Tables**) in this study implies that degradation of lipids and carboxylic acids can potentially be used as carbon sources for *R*. *solani* prior to onset of necrosis when nutrients are limited. These genes were also induced in *R*. *solani* in the presence of *Serratia* species [47], demonstrating their importance in stress tolerance and defense or attack during interaction with biotic stresses.

The γ-aminobutyric acid (GABA) shunt pathway can be used by fungi to obtain alternative carbon and nitrogen sources [75] and is derived by conversion of glutamate to GABA via the enzyme glutamate decarboxylase. A putative gene encoding *GLUTAMATE DECARBOXYLASE* (*GAD;* ELU39983) was highly up-regulated at onset and 24 h.p.o. of necrosis (**S5 and S6 Tables**), with no differences between the two time points in samples infecting soybean. Genes involved in the GABA shunt were also differentially expressed when a *R*. *solani* strain belonging to AG1-IB was grown in the presence of lettuce root exudates [57], suggesting that GABA utilization may have a strong role in the invasion and establishment of *R*. *solani* in its host. The possibility that fungi are able to utilize plant-derived GABA as a carbon and nitrogen source for pathogenicity during plant host invasion was suggested in the case of tomato-*Cladosporium fulvum* [76]. Interestingly, decreases in the abundance soybean-derived GABA during the interaction between *R*. *solani* AG1-IA and soybean compared to uninfected controls was reported [77]. Together these findings indicate that pathogenic fungi may alter plant leaf physiology in order to gain access to nitrogen and carbon sources. GABA in fungi can also act as a signalling molecule for the induction of plant cell wall degrading enzymes [78, 79]. The large increase in *GLUTAMATE DECARBOXYLASE* at onset of necrosis suggests that GABA may also act as a signalling molecular in *R*. *solani*.

## Conclusions

We have conducted the first comprehensive high throughput RNA sequencing study of *R*. *solani* AG1-IA (strain ROS-2A4), the causal agent of Rhizoctonia foliar blight, at two different infection stages of soybean. The differential expression of *R*. *solani* AG1-IA transcripts provided us with insights on the shifts in gene expression of major primary and secondary metabolic processes and the activation of defence and attack mechanisms. The list of candidate genes associated with defence and attack identified in this study might provide a basis for future identification of fungal pathogenicity genes, as well as provide a foundation for targeted control methods and novel strategies for the development of RFB resistant soybean lines.

## Supporting information

S1 Table KEGG pathway E.C. numbers associated with genes expressed at onset of necrosis. (XLSX)

S2 Table KEGG pathway E.C. numbers associated with genes expressed 24 h.p.o. of necrosis. (XLSX)

S3 Table qRT-PCR primers and thermocycling conditions. (DOCX)

S4 Table Summary of RNA-seq read numbers and alignment rates. (DOCX)

S5 Table Differentially expressed genes during *Rhizoctonia*-soybean interactions at onset of necrosis. (XLSX)

S6 Table Differentially expressed genes during *Rhizoctonia*-soybean interactions 24 hours post-onset of necrosis. (XLSX)

S7 Table Differential gene expression between *R*. *solani* genes at onset and 24 h.p.o. of necrosis. (XLSX)

S8 Table Top 20 up-regulated genes of *Rhizoctonia solani* at onset and 24 hours post-onset of necrosis during soybean interactions. (DOCX)

S9 Table Top 20 down-regulated genes of *Rhizoctonia solani* at onset and 24 hours post-onset of necrosis during soybean interactions. (DOCX)

S1 Fig KEGG pathway annotations that were unique for each *Rhizoctonia solani*-soybean interaction time point. (DOCX)
