# Supplementary material for: The transcriptional landscape of Rhizoctonia solani AG1-IA during infection of soybean as defined by RNA-seq
Source: PLoS One. 2017 Sep 6;12(9):e0184095. doi: 10.1371/journal.pone.0184095 (PMC5587340; doi:10.1371/journal.pone.0184095)
Supplement: S3 Table — (DOCX) [file pone.0184095.s003.docx]

**S3 Table**. **qPCR primers and thermocycling conditions**

| Pathway | Gene Locus | Annotation | Primer name | Primer set (5’-3’) | Product Size (bp) | Annealing temp. (˚C) | Final primer concentration (µM) |
| --- | --- | --- | --- | --- | --- | --- | --- |
| Carbohydrate  and Carbon metabolism | ELU42665 | Alpha-amylase (*AMY*) | RS_AMY_F  RS_AMY_R | AAGCGAAGCTGGGAACAAT  TAATATCCGCGAGTTGGTTGAC | 268 | 53 | 0.25 |
|  | ELU38592 | Chitin deacetylase (*CDC*) | RS_CDC_F | GCATGACGTAGTCCTAAGAAGG | 207 | 52 | 0.25 |
|  |  |  | RS_CDC_R | CTCGTTCCCGTCGCTATATTC |  |  |  |
|  | ELU42868 | Beta-glucosidase (*BGLUC*) | RS_BGLUC_F | TGGTTCGCAGACCCTATTTAC | 201 | 52 | 0.25 |
|  |  |  | RS_BGLUC_R | GGTGTACTGGACGTTTCCTT |  |  |  |
|  | ELU37123 | Laccase precursor (*LAC*) | RS_LAC_F2 | CCAAGGGCACGGCTATAAA | 122 | 53 | 0.25 |
|  |  |  | RS_LAC_R | CATCTCGAACGATAGGGACAAG |  |  |  |
|  | ELU38450 | Formate dehydrogenase (*FDH*) | RS_FDH_F | CCAAGAAGAACGAGCAGAAATG | 158 | 52 | 0.25 |
|  |  |  | RS_FDH_R | TCCAGCTGCCTTGTACGACCT |  |  |  |
|  | ELU41358 | Glycogen synthase (*GCS*) | RS_GCS_F | CTTATCCTGATGCCTTCGGTG | 187 | 52 | 0.25 |
|  |  |  | RS_GCS_R | GGCCATACTTGACCCTTGTAATC |  |  |  |
| Redox  Reactions | ELU41063 | NADH oxidase (*NOX*) | RS_NOX_F | GTGTCGAATTTCAGGCGAAAG | 244 | 52 | 0.25 |
|  |  |  | RS_NOX_R | CGGAATCCACCGGTAACATAA |  |  |  |
|  | ELU40841 | Pyridoxal-dependent decarboxylase (*PDX*) | RS_PDX_F | GAACAACCAAGCATTACTCGTG | 80 | 53 | 0.25 |
|  |  |  | RS_PDX_R | GACCGGGACGTCAATGATATG |  |  |  |
|  | ELU39168 | Thiamine biosynthesis (*THI*) | RS_THI_F | TAATATCCGCGAGTTGGTTGAC | 136 | 52 | 0.25 |
|  |  |  | RS_THI_R | CCAAGCCTCTTCGAGTAGTTAG |  |  |  |
|  | ELU45264 | Glutathione-S-transferase (*GST*) | RS_GST_F | GGATGCTAAGCTCGATGGATAC | 190 | 52 | 0.2 |
|  |  |  | RS_GST_R | GGATGCTAAGCTCGATGGATAC |  |  |  |
|  | ELU42795 | Cu/Zn superoxide dismutase (*SOD*) | RS_SOD_F | GCAAGATCACTGGCCTAACA | 195 | 52 | 0.25 |
|  |  |  | RS_SOD_R | CAACTTTGGATTCGCCATTCG |  |  |  |
| Detoxification | ELU36963 | Cytochrome P450 monooxygenase pc-3 (*P450*) | RS_P450_F | CAACCTATCGCAGTGGACTTT | 117 | 52 | 0.25 |
|  |  |  | RS_P450_R | GTGAGGATAGGGAAGGGTAGAA |  |  |  |
|  | ELU43748 | ABC transporter (*ABC*) | RS_ABC_F | AGCATTTGGTGGTGATGTAGAA | 224 | 52 | 0.25 |
|  |  |  | RS_ABC_R | CCAGGCTCTTTGCGATGTAATA |  |  |  |
| DNA  modification | ELU43810^a^ | Histone 3 (*H3*) | RS_H3_F | CTTCCAATCATCGGCAGTCCTC | 76 | 52 | 0.2 |
|  |  |  | RS_H3_R | ATTGGTATCTTCGAACAAAGACACGAG |  |  |  |

^a^Housekeeping gene
