# Supplementary material for: The transcriptional landscape of Rhizoctonia solani AG1-IA during infection of soybean as defined by RNA-seq
Source: PLoS One. 2017 Sep 6;12(9):e0184095. doi: 10.1371/journal.pone.0184095 (PMC5587340; doi:10.1371/journal.pone.0184095)
Supplement: S4 Table — (DOCX) [file pone.0184095.s004.docx]

**S4 Table.** Summary of RNA-seq read numbers and alignment rates

| **Sample^a^** | **Total input reads** | **Left mapped reads** | **Right mapped reads** | **Total aligned paired reads** | **Concordant alignment rate** |
| --- | --- | --- | --- | --- | --- |
| CO_R1 | 13,951,596 | 9,327,490 (66.9%) | 9,554,656 (68.5%) | 8,173,617 | 58.6% |
| CO_R2 | 12,298,549 | 8,350,660 (67.9%) | 8,579,948 (69.8%) | 7,299,480 | 59.4% |
| CO_R3 | 10,853,548 | 7,652,711 (70.5%) | 7,935,010 (73.1%) | 6,662,964 | 61.4% |
| C24_R1 | 12,294,014 | 8,300,723 (67.5%) | 8,499,282 (69.1%) | 7,275,077 | 59.2% |
| C24_R2 | 11,523,256 | 7,579,459 (65.8%) | 7,818,303 (67.8%) | 6,655,490 | 57.8% |
| C24_R3 | 10,691,097 | 7,348,586 (68.7%) | 7,696,319 (72.0%) | 6,354,395 | 59.4% |
| IO_R1 | 13,516,690 | 9,735,594 (72.0%) | 9,911,165 (73.3%) | 8,645,369 | 64.0% |
| IO_R2 | 11,695,661 | 8,275,389 (70.8%) | 8,433,003 (72.1%) | 7,243,702 | 61.9% |
| IO_R3 | 10,209,180 | 7,206,513 (70.6%) | 7,353,903 (72.0%) | 6,295,991 | 61.7% |
| I24_R1 | 15,386,553 | 11,508,108 (74.8%) | 11,719,118 (76.2%) | 10,237,803 | 66.5% |
| I24_R2 | 12,582,972 | 9,502,256 (75.5%) | 9,663,459 (76.8%) | 8,432,507 | 67.0% |
| I24_R3 | 13,023,270 | 9,565,554 (73.4%) | 9,730,816 (74.7%) | 8,567,603 | 65.8% |

^a^Sample labeling: C, control samples grown on ¼ strength PDA; I, *R. solani*-soybean interaction samples; O, onset of necrosis; 24, 24 hours post-onset of necrosis; R1,R2,R3, replicates 1 through 3.
