# Supplementary material for: The transcriptional landscape of Rhizoctonia solani AG1-IA during infection of soybean as defined by RNA-seq
Source: PLoS One. 2017 Sep 6;12(9):e0184095. doi: 10.1371/journal.pone.0184095 (PMC5587340; doi:10.1371/journal.pone.0184095)
Supplement: S8 Table — (DOCX) [file pone.0184095.s008.docx]

**S8 Table**. Top 20 up-regulated genes of *Rhizoctonia solani* at onset and 24 hours post-onset of necrosis during soybean interactions

| **Gene ID** | **Putative function** | **Fold change** |
| --- | --- | --- |
| **Onset** |  |  |
| ELU41255 | S1 P1 nuclease domain-containing | 164.69 |
| ELU42021 | Succinate:fumarate antiporter | 138.59 |
| ELU42599 | Acetyltransferase (GNAT) family domain-containing | 119.70 |
| ELU40632 | Lysine ornithine N-monooxygenase | 100.28 |
| ELU42395 | Ribonuclease domain-containing | 64.97 |
| ELU36836 | Serine carboxypeptidase domain-containing | 60.10 |
| ELU39983 | Glutamate decarboxylase | 51.06 |
| ELU37627 | Protein ura1 | 43.93 |
| ELU42975 | Methyltransferase domain-containing | 38.55 |
| ELU36224 | Hemopexin domain-containing | 36.84 |
| ELU38977 | Methyltransferase domain-containing | 35.39 |
| ELU42372 | Malate synthase | 33.20 |
| ELU42297 | NADH:flavin oxidoreductase NADH oxidase | 32.98 |
| ELU42027 | Acyltransferase family domain-containing | 31.18 |
| ELU43846 | AlcB domain-containing | 29.73 |
| ELU42133 | Ribonuclease t2 family domain-containing | 29.13 |
| ELU40590 | GPR1 FUN34 yaaH family domain-containing | 28.41 |
| ELU42127 | Major facilitator superfamily | 27.04 |
| ELU39168 | Thiamine biosynthesis | 25.58 |
| ELU35711 | Aldedh domain-containing | 24.57 |
| **24 h.p.o.^A^** |  |  |
| ELU42975 | Methyltransferase domain-containing | 161.60 |
| ELU37705 | Betaine lipid synthetase | 86.54 |
| ELU42297 | NADH:flavin oxidoreductase NADH oxidase | 73.01 |
| ELU41329 | Glycosyl hydrolase family 10 domain-containing | 53.36 |
| ELU40684 | Aldo-keto reductase family domain-containing | 48.74 |
| ELU40694 | Glucanase | 47.70 |
| ELU41328 | Glycosyl hydrolase family 10 domain-containing | 44.19 |
| ELU36802 | 1,4-Beta-D-glucan-cellobiohydrolase | 33.75 |
| ELU45593 | Cytoplasmic tRNA 2-thiolation 1 | 32.79 |
| ELU42849 | Sodium:inorganic phosphate symporter | 27.62 |
| ELU39983 | Glutamate decarboxylase | 27.36 |
| ELU36357 | Exocellobiohydrolase | 26.19 |
| ELU45366 | MFS multidrug transporter | 24.36 |
| ELU37146 | Short chain dehydrogenase domain-containing | 24.26 |
| ELU35955 | Phospho-2-dehydro-3-deoxyheptonate aldolase | 20.24 |
| ELU41063 | NADH oxidase | 19.62 |
| ELU37627 | Protein ura1 | 18.54 |
| ELU41255 | S1 P1 nuclease domain-containing | 18.30 |
| ELU43022 | Ferrate chelate reductase | 17.64 |
| ELU41805 | Sim15 | 17.17 |

^A^h.p.o.: hours post-onset of necrosis
