# Supplementary material for: The transcriptional landscape of Rhizoctonia solani AG1-IA during infection of soybean as defined by RNA-seq
Source: PLoS One. 2017 Sep 6;12(9):e0184095. doi: 10.1371/journal.pone.0184095 (PMC5587340; doi:10.1371/journal.pone.0184095)
Supplement: S9 Table — (DOCX) [file pone.0184095.s009.docx]

**S9 Table.** Top 20 down-regulated genes of *Rhizoctonia solani* at onset and 24 hours post-onset of necrosis during soybean interactions

| **Gene ID** | **Putative function** | **Fold change** |
| --- | --- | --- |
| **Onset** |  |  |
| ELU43994 | Tyrosinase domain-containing | -437.64 |
| ELU39832 | Tyrosinase domain-containing | -145.99 |
| ELU42092 | OPT oligopeptide transporter family | -136.96 |
| ELU42795 | Coppper zinc superoxide dismutase domain-containing | -94.67 |
| ELU37197 | Plastocyanin-like domain-containing | -79.29 |
| ELU36581 | Tyrosinase domain-containing | -56.94 |
| ELU37123 | Laccase precursor | -55.36 |
| ELU39641 | CVNH domain-containing | -45.27 |
| ELU43602 | Copper radical oxidase | -41.60 |
| ELU41627 | Histone domain-containing | -31.26 |
| ELU39298 | Mannoprotein | -30.63 |
| ELU36531 | DNA-J related domain-containing | -29.01 |
| ELU36633 | Copper radical oxidase | -28.09 |
| ELU41603 | Histone H3 | -26.04 |
| ELU38592 | Chitin deacetylase | -25.64 |
| ELU43031 | Glycoside hydrolase family 16 | -24.89 |
| ELU44549 | Carboxylic transporter | -24.86 |
| ELU42320 | Glycosyl hydrolase family 61 domain-containing | -24.48 |
| ELU39683 | Glycophorin A domain-containing | -23.91 |
| ELU45794 | Polysaccharide deacetylase domain-containing | -21.39 |
| **24 h.p.o.^A^** |  |  |
| ELU37080 | Ricin-type beta-trefoil lectin domain-containing | -1010.40 |
| ELU40841 | Pyridoxal-dependent decarboxylase conserved domain-containing | -758.50 |
| ELU37197 | Plastocyanin-like domain-containing | -605.51 |
| ELU42209 | Ricin-type beta-trefoil lectin domain-containing | -536.69 |
| ELU36143 | Group II decarboxylase | -234.04 |
| ELU39832 | Tyrosinase domain-containing | -195.98 |
| ELU43994 | Tyrosinase domain-containing | -169.98 |
| ELU42242 | Peroxidase family 2 domain-containing | -149.20 |
| ELU41055 | Ferritin-like domain-containing | -145.26 |
| ELU36581 | Tyrosinase domain-containing | -126.75 |
| ELU35548 | Fungalysin metallopeptidase (M36) domain-containing | -104.40 |
| ELU37123 | Laccase precursor | -96.70 |
| ELU38273 | Rare lipo A()-like double-psi beta-barrel domain-containing | -89.78 |
| ELU40436 | Short-chain dehydrogenase | -76.91 |
| ELU40556 | Mannosyltransferase domain-containing | -72.25 |
| ELU42795 | Copper zinc superoxide dismutase domain-containing | -61.10 |
| ELU43602 | Copper radical oxidase | -57.71 |
| ELU38231 | Chorismate mutase | -53.33 |
| ELU39641 | CVNH domain-containing | -45.72 |
| ELU37059 | Rieske [2Fe-2S] domain-containing | -45.42 |

^A^h.p.o.: hours post-onset of necrosis
