# Supplementary material for: The transcriptional landscape of Rhizoctonia solani AG1-IA during infection of soybean as defined by RNA-seq
Source: PLoS One. 2017 Sep 6;12(9):e0184095. doi: 10.1371/journal.pone.0184095 (PMC5587340; doi:10.1371/journal.pone.0184095)
Supplement: S1 Fig — (DOCX) [file pone.0184095.s010.docx]

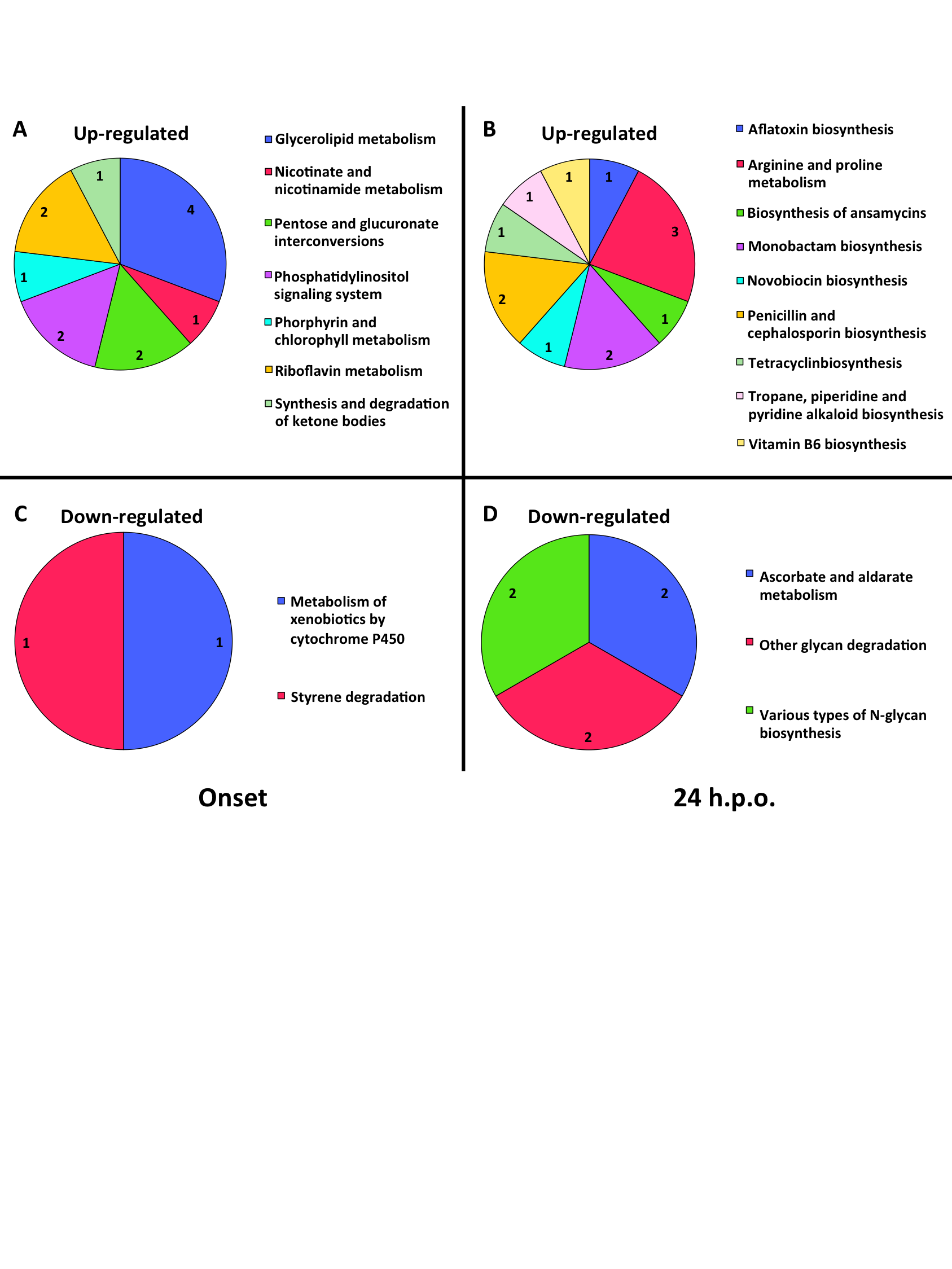


**S1 Fig. KEGG pathway annotations that were unique for each *Rhizoctonia solani*-soybean interaction time point.** Unique KEGG pathways (**A**) up-regulated at the onset of necrosis, (**B**) up-regulated 24 h.p.o. of necrosis, (**C**) down-regulated at the onset of necrosis, (**D**) down-regulated 24 hours h.p.o. of necrosis (*n*=3 per treatment). Numbers represent the number of differentially expressed genes with fold change values +/- 3 detected for each KEGG pathway.
